# Hyperglycemia and glucose variability are associated with worse survival in mechanically ventilated COVID-19 patients: the prospective Maastricht Intensive Care Covid Cohort

**DOI:** 10.1186/s13098-023-01228-1

**Published:** 2023-12-06

**Authors:** Thijs T.W. van Herpt, Frank van Rosmalen, Hendrica P.M.G. Hulsewé, Anouk N.A. van der Horst-Schrivers, Mariëlle Driessen, Robin Jetten, Noortje Zelis, Bastiaan E. de Galan, Sander M.J. van Kuijk, Iwan C.C. van der Horst, Bas C.T. van Bussel

**Affiliations:** 1 Department of Intensive Care Medicine, Maastricht University Medical Centre +, Debyelaan 25, 6229 HX Maastricht, the Netherlands; 2 Department of Emergency Medicine, Maastricht University Medical Centre +, Maastricht, The Netherlands; 3 Department of Endocrinology, Maastricht University Medical Centre+, Maastricht, The Netherlands; 4 Department of Internal Medicine, Maastricht University Medical Centre +, Maastricht, The Netherlands; 5 Department of Clinical Epidemiology and Medical Technology Assessment, Maastricht University Medical Centre+, Maastricht, The Netherlands; 6 Cardiovascular Research Institute Maastricht (CARIM), Maastricht University, Maastricht, The Netherlands; 7 Care and Public Health Research Institute (CAPHRI), Maastricht University, Maastricht, the Netherlands

**Keywords:** Prognosis, Glycemic variability, Hyperglycemia, COVID-19, Mortality, Prognosis, Sars-CoV-2 ARDS

## Abstract

**Background:**

Data on hyperglycemia and glucose variability in relation to diabetes mellitus, either known or unknown in ICU-setting in COVID-19, are scarce. We prospectively studied daily glucose variables and mortality in strata of diabetes mellitus and glycosylated hemoglobin among mechanically ventilated COVID-19 patients.

**Methods:**

We used linear-mixed effect models in mechanically ventilated COVID-19 patients to investigate mean and maximum difference in glucose concentration per day over time. We compared ICU survivors and non-survivors and tested for effect-modification by pandemic wave 1 and 2, diabetes mellitus, and admission HbA1c.

**Results:**

Among 232 mechanically ventilated COVID-19 patients, 21.1% had known diabetes mellitus, whereas 16.9% in wave 2 had unknown diabetes mellitus. Non-survivors had higher mean glucose concentrations (ß 0.62 mmol/l; 95%CI 0.20–1.06; ß 11.2 mg/dl; 95% CI 3.6–19.1; *P* = 0.004) and higher maximum differences in glucose concentrations per day (ß 0.85 mmol/l; 95%CI 0.37–1.33; ß 15.3; 95%CI 6.7–23.9; *P* = 0.001). Effect modification by wave, history of diabetes mellitus and admission HbA1c in associations between glucose and survival was not present. Effect of higher mean glucose concentrations was modified by pandemic wave (wave 1 (ß 0.74; 95% CI 0.24–1.23 mmol/l) ; (ß 13.3; 95%CI 4.3–22.1 mg/dl)) vs. (wave 2 (ß 0.37 (95%CI 0.25–0.98) mmol/l) (ß 6.7 (95% ci 4.5–17.6) mg/dl)).

**Conclusions:**

Hyperglycemia and glucose variability are associated with mortality in mechanically ventilated COVID-19 patients irrespective of the presence of diabetes mellitus.

## Background

Diabetes mellitus is a comorbid condition often reported in Severe Acute Respiratory Syndrome Coronavirus 2 (SARS-CoV-2, COVID-19) [1, 2] and associated with poor prognosis [3, 4]. Even among COVID-19 patients without a known history of diabetes mellitus, increased glucose concentrations both at and during admission [5–7] and, to a lesser extent, elevated glycated hemoglobin (HbA1c) levels [8, 9] are associated with worse disease outcome. In general, hyperglycemia [10, 11] and high glucose variability [12–19] worsen the prognosis of patients admitted to the intensive care unit (ICU) with diabetes mellitus modulating this effect [20, 21]. In COVID-19, the mechanisms through which dysglycemia affects outcome in the ICU is still unknown, although unknown diabetes mellitus status and treatment with dexamethasone may play a role [22–24].

Identification of the factors leading to dysglycemia in COVID-19 patients and dismal prognosis is critical to improve glucose control by targeted monitoring. Whereas recent work has focused on glucose concentrations [6, 7, 9] and HbA1c [8, 9, 25] as static parameters, it should be acknowledged that glucose concentrations constantly fluctuate. This may impact the course of the disease and vice versa, necessitating (semi-) continuous monitoring. Acute inflammation by infectious diseases, as well as steroid treatment, affects glucose metabolism. Therefore, it is important to investigate the impact of glucose variability on the outcome in COVID-19 patients. Since data on hyperglycemia and glucose variability in the ICU-setting in COVID-19 are scarce [8, 17, 26, 27] we aimed, in a comprehensive observational prospective study, to investigate the association between daily glucose concentrations and the survival of mechanically ventilated patients with COVID-19. Our hypotheses were that: (1) higher mean glucose concentrations and greater daily glucose variability are independently associated with worse survival; (2) those associations are stronger in steroid-treated patients and in patients with diabetes mellitus, whether known or unknown history.

## Methods

### Study design and population

The Maastricht Intensive Care COVID (MaastrICCht) cohort study design has been described more extensively elsewhere [28–31]. Briefly, this prospective cohort study included patients admitted to the Intensive Care of the Maastricht University Medical Centre+ (Maastricht UMC+), a tertiary care university teaching hospital in the southern part of the Netherlands. The local institutional review board (Medisch Ethische Toetsingscomissie (METC) 2020 − 1565/ 300,523) of the Maastricht UMC + approved the study, which was performed based on the declaration of Helsinki. Despite the challenging times of the COVID-19 pandemic [32], all patients and their families provided complete informed consent for the utilization of the collected data and storage of leftover serum samples for critical COVID-19 research purposes. The study has been registered in the Netherlands Trial Register (registration number NL8613). This study included all participants with respiratory insufficiency requiring mechanical ventilation and at least one positive PCR test for SARS-CoV-2 and/or a chest CT scan strongly suggestive of SARS-CoV-2 infection, based on a COVID-19 Reporting and Data System (CORADS)-score of 4–5 [33]. Participants were followed from the moment of intubation until the primary outcome (death during ICU admission or discharge from the ICU). Clinical, physiological, and laboratory variables were collected using a predefined study protocol described elsewhere [28]. For the present study, participants were included based on the day of the start of mechanical ventilation/intubation in wave 1 from March 15th, 2020, until July 2020, and in wave 2 from October 2020 until March 23th, 2021. Thus data from the first and second waves were included. During the first wave, patients were intubated according to early Dutch intensive care guidelines, as there were concerns about the virus spread using other modes of oxygen or ventilator support [34]. However, since accumulating evidence shows its safety [35] high-flow nasal oxygen was applied in the second wave onwards. As we aimed to investigate the development of variables over time, like in previous reports [30, 36], we included intubated and mechanically ventilated patients and set intubation as day one. This makes patients at inclusion to be assumed at similar time-points of disease and severity during their COVID-19 disease, i.e., an inception cohort. In addition, this allows us to take all observations into account over time, also if a patient is transported after intubation from another ICU, facilitating the investigation of the development of variables over time [29, 30, 36].

### Diabetes Mellitus, glucose, insulin, and HbA1c

Diabetes mellitus was defined as a reported history of diabetes mellitus and/or the use of glucose-lowering medication. As diabetes mellitus may drive severe COVID-19 disease [3, 4] and might be undetected, we measured HbA1c. HbA1c was prospectively measured in the cohort from wave 2 onwards, as dexamethasone became the standard of care [37] to improve the monitoring of patients at risk of dysglycemia. HbA1c was defined as high when equal to or above 48 mmol/mol (6.5%), aligning with the diagnostic criteria for a diagnosis of diabetes mellitus according to the guidelines of the American Diabetes Association [38] and the World Health Organization [39]. The comprehensive cohort data were enriched with serial glucose variables extracted from the electronic patient files by automation. Glucose measurements were conducted following standards of care and obtained from blood gas analysis, point-of-care testing, and venous sampling procedures. The prescription and administration of insulin was carried out by the hospital’s standard medical personnel in accordance with established care protocols. Glucose data were expressed as two variables: mean glucose concentration in mmol/l and mg/dl per day and maximum glucose difference (max glucose - min glucose) in mmol/l and mg/dl per day. We have chosen maximum difference in glucose concentration since it is a straight-forward measure and has a strong predictive ability in ICU patients [40]. Nasal-gastric feeding was protocolized care in mechanically ventilated patients and accompanied by continuous insulin therapy. Additionally, we also collected data on continuous insulin dosing and summarized this as the total insulin dose per day (in total units administered).

### Ascertainment of comorbidities and mortality

As previously described, data on comorbidities and ICU discharge or death were collected using an electronic case report form (eCRF) [28]. Briefly, information on the presence of comorbidities (liver disease, chronic lung disease, and chronic kidney disease was recorded when diagnosed by a medical specialist) was retrieved from medical records. Next, information on cardiovascular risk factors was extracted from each patient’s medical file. We defined cardiovascular risk as a history of hypertension, previous myocardial infarction, cerebrovascular disease, peripheral vascular disease, history of smoking, and/or coronary artery disease. Finally, ICU discharge or death was extracted from the medical files.

### Statistical analyses and reporting

The manuscript was written following the Strengthening the Reporting of OBservational studies in Epidemiology (STROBE) guideline [41]. Automatic data extraction was performed using Matlab 2019b. Data were analyzed using R version 4.1.1. Continuous variables are expressed as mean +/- SD, or median with 25th – 75th percentile. For illustrative purpose, we divided the sample into six predefined strata: patients included during the first wave of the pandemic (HbA1c not measured) without [1] and with [2] a known history of diabetes mellitus; patients included during the second wave of the pandemic without [3] and with [4] a known history of diabetes mellitus with low HbA1c, and patients included during the second wave of the pandemic without [5] and with [6] a known history of diabetes mellitus with high HbA1c. For these six strata, serial glucose concentrations per day from intubation onwards are shown. In addition, mean insulin units per day are shown.

The associations between glycemic parameters (mean glucose, maximal glucose difference, and additionally insulin use per day) and ICU mortality were estimated. In order to do so, the full cohort was categorized into survivors and non-survivors. We used linear-mixed effect models to investigate the development of mean glucose concentration per day over time, to investigate the development of maximum difference in glucose concentration per day over time, and compared ICU survivors and non-survivors. First, model 1 consisted of crude models whereas model 2 included crude models adjusted for covariates as age, sex, body mass index (BMI), Acute Physiology and Chronic Health Evaluation-score II (APACHE II score), chronic kidney, pulmonary and liver diseases, and cardiovascular risk factors. Next, effect-modification by pandemic wave (wave one without protocolized dexamethasone therapy and wave 2 with protocolized dexamethasone therapy), reported history of diabetes mellitus and low vs. high HbA1c (in wave 2 only) were investigated. Additionally, the development of total insulin units for ICU survivors and non-survivors per day over time was investigated. We considered a p-value < 0.05 and a p-value for interaction < 0.10 statistically significant.

## Results

**Fig. 1:**
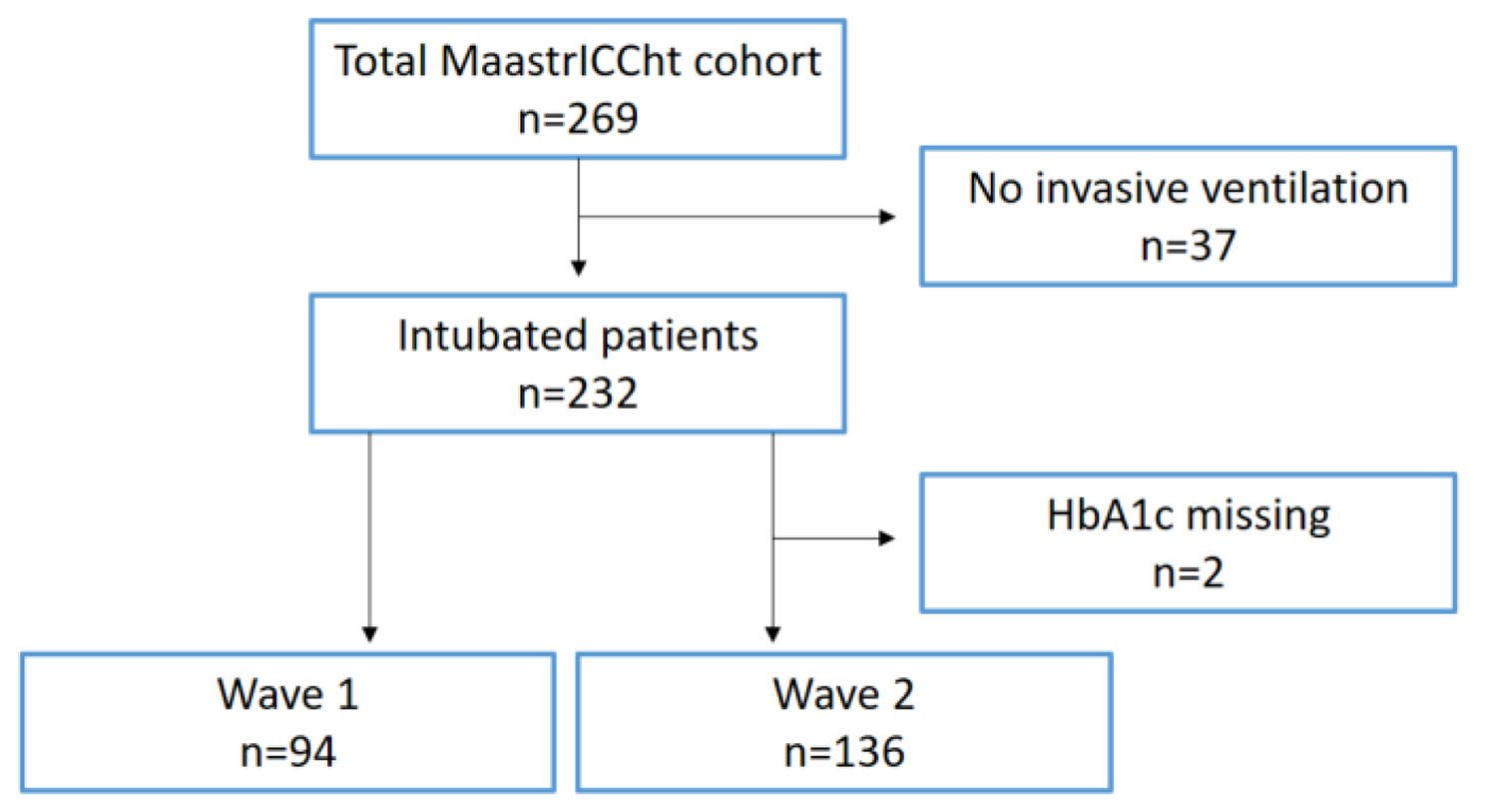
Selection of study participants. Wave 1 = March 2020 – July 2020; Wave 2 = October 2020 – March 2021

During the inclusion period, 269 patients were screened (Figure 1), 37 (13.8%) of whom did not receive invasive ventilation, so that 232 intubated and mechanically ventilated patients were included (wave 1, n = 94; wave 2, n = 138; Table 1).

All patients admitted to the Intensive Care Unit (ICU) were admitted for their initial hospitalization, with no instances of readmission. Diabetes mellitus was present in 49 patients (21.1%). In wave 2, 23 patients (16.9%) had high HbA1c (Above 48mmol/mol (6.5%)) without a previous diagnosis of diabetes mellitus. During a median ICU length of stay of 14 [8-22.5] days, glucose was measured 4 [3–5] times daily, leading to a total of 19,191 measurements.

**Table 1:** Baseline characteristics for the total sample and pre-specified groups

	Total	Group 1 (n = 80)	Group 2 (n = 14)	Group 3 (n = 78)	Group 4 (n = 2)	Group 5 (n = 23)	Group 6 (n = 33)
***Predefined strata characteristics***:							
Wave		**1**	**1**	**2**	**2**	**2**	**2**
HbA1c status		**-**	**-**	**Low**	**Low**	**High**	**High**
Previously diagnosed diabetes mellitus		**No**	**Yes**	**No**	**Yes**	**No**	**Yes**
***Admission characteristics***:							
Age	66 (58–73)	66 (56–73)	73 (66–75)	65 (58–72)	73.5 (70–77)	64 (57–71)	64 (59–70)
Gender (female)	55 (23.7%)	20 (25.0%)	1 (7.1%)	26 (33.3%)	0 (0.0%)	3 (13.0%)	4 (12.1%)
BMI (kg/m^2^)	27.7 (25-30.7)	27 (24.9–29.6)	28.2 (25.9–30.9)	26.9 (24.8–29.2)	29.1 (24.6–33.6)	28.7 (26.5–32.2)	28.9 (26.8–33.5)
APACHE II score	15 (13–18)	16 (13–18)	15 (14–19)	14 (13–16)	18.5 (17–20)	16 (13–18)	14 (11-17.5)
HbA1c (mmol/mol)	50.7 (15.8)	NA	NA	41.1 (3.9)	41.0 (2.8)	52.8 (5.3)	72.9 (15.7)
HbA1c (%)	6.9 (2.1)	NA	NA	5.6 (0.5)	5.6 (0.5)	7.2 (0.7)	9.9 (2.1)
***Chronic health conditions***:							
Diabetes mellitus	49 (21.1%)	0 (0.0%)	14 (100.0%)	0 (0.0%)	2 (100.0%)	0 (0.0%)	33 (100.0%)
Chronic kidney disease	6 (2.6%)	1 (1.3%)	1 (7.1%)	1 (1.3%)	0 (0.0%)	0 (0.0%)	3 (9.1%)
Chronic liver disease	1 (0.4%)	0 (0.0%)	1 (7.1%)	0 (0.0%)	0 (0.0%)	0 (0.0%)	0 (0.0%)
Chronic lung disease	37 (15.9%)	5 (6.3%)	3 (21.4%)	18 (23.1%)	1 (50.0%)	4 (17.4%)	6 (18.2%)
***Cardiovascular risk factors***:							
Hypertension	79 (34.1%)	23 (28.7%)	10 (71.4%)	16 (20.5%)	0 (0.0%)	9 (39.1%)	19 (57.6%)
Myocardial infarction	29 (12.5%)	4 (5.0%)	2 (14.3%)	11 (14.1%)	0 (0.0%)	3 (13.0%)	9 (27.3%)
Current smoking	13 (5.6%)	5 (6.3%)	2 (14.3%)	5 (6.4%)	0 (0.0%)	0 (0.0%)	1 (3.0%)
Coronary artery disease	18 (7.8%)	6 (7.5%)	3 (21.4%)	4 (5.1%)	0 (0.0%)	1 (4.3%)	4 (12.1%)
***ICU stay characteristics***:							
Immunosuppressive medication	16 (6.9%)	2 (2.5%)	0 (0.0%)	9 (11.5%)	0 (0.0%)	3 (13.0%)	2 (6.1%)
Length of stay (days)	14 (8–23)	16 (8–28)	11.5 (4–18)	13 (8–21)	11 (8–14)	14 (10–31)	13 (8–29)

Data are means +/- SD or count (%) as appropriate for the total population and pre-specified groups. Wave 1 = March 2020 – July 2020; Wave 2 = October 2020 – March 2021). Group 1: wave 1, no previous diagnosis of diabetes mellitus. Group 2: wave 1, previously diagnosed diabetes mellitus. Group 3: wave 2, no previous diagnosis of diabetes mellitus, low HbA1c. Group 4: wave 2, previously diagnosed diabetes mellitus, low HbA1c. Group 5: wave 2, no previous diagnosis of diabetes mellitus, high HbA1c. Group 6: wave 2, previously diagnosed diabetes mellitus, high HbA1c. In wave 1, no HbA1c was measured. High HbA1c is defined as above 48mmol/mol (6.5%)

**Fig. 2:**
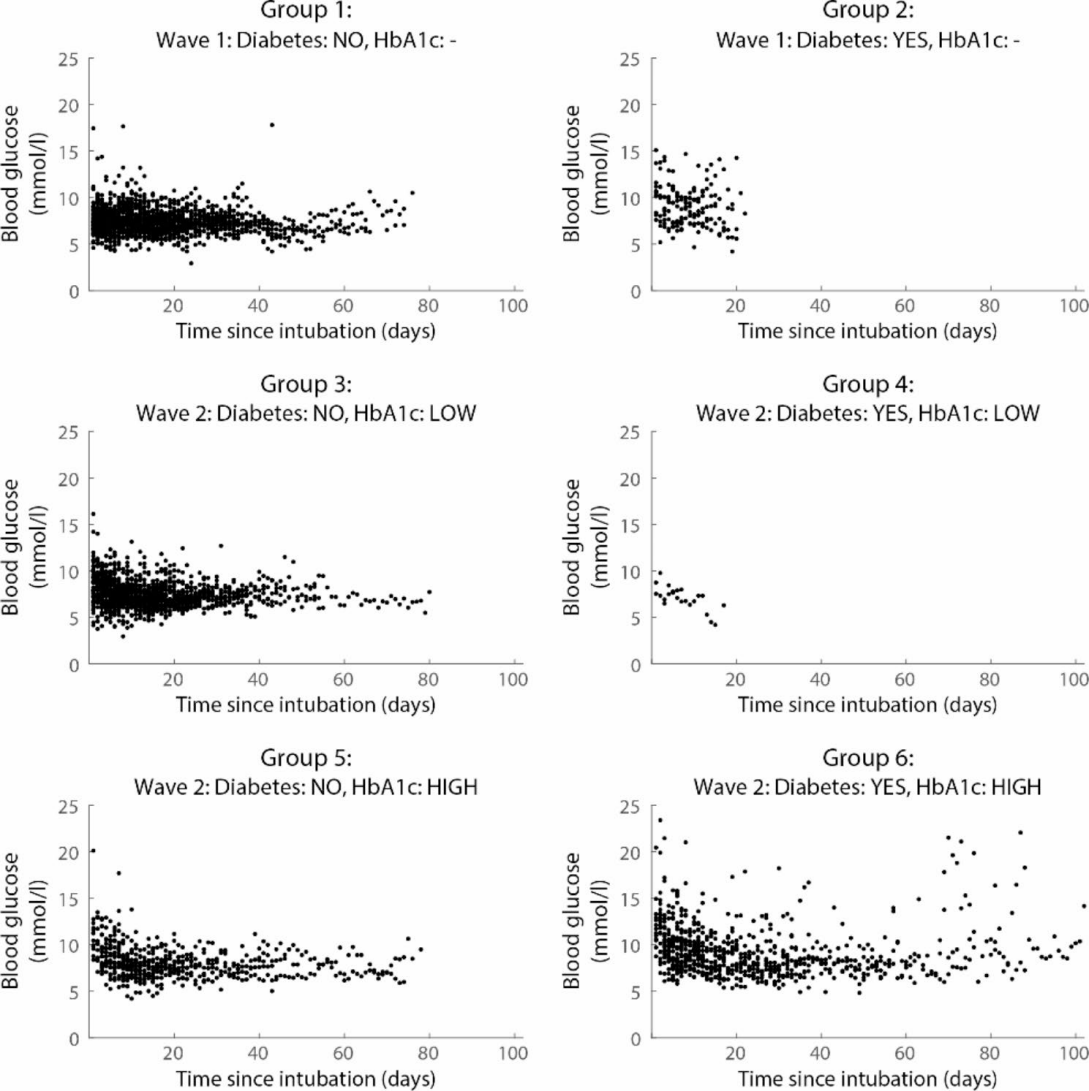
Descriptive data of mean daily glucose concentrations over time from intubation onwards, according to predefined strata. Group 1: wave 1, no previous diagnosis of diabetes mellitus. Group 2: wave 1, previously diagnosed diabetes mellitus. Group 3: wave 2, no previous diagnosis of diabetes mellitus, low HbA1c. Group 4: wave 2, previously diagnosed diabetes mellitus, low HbA1c. Group 5: wave 2, no previous diagnosis of diabetes mellitus, high HbA1c. Group 6: wave 2, previously diagnosed diabetes mellitus, high HbA1c. In wave 1, no HbA1c was measured. High HbA1c is defined as above 48mmol/mol (6.5%)

**Fig. 3:**
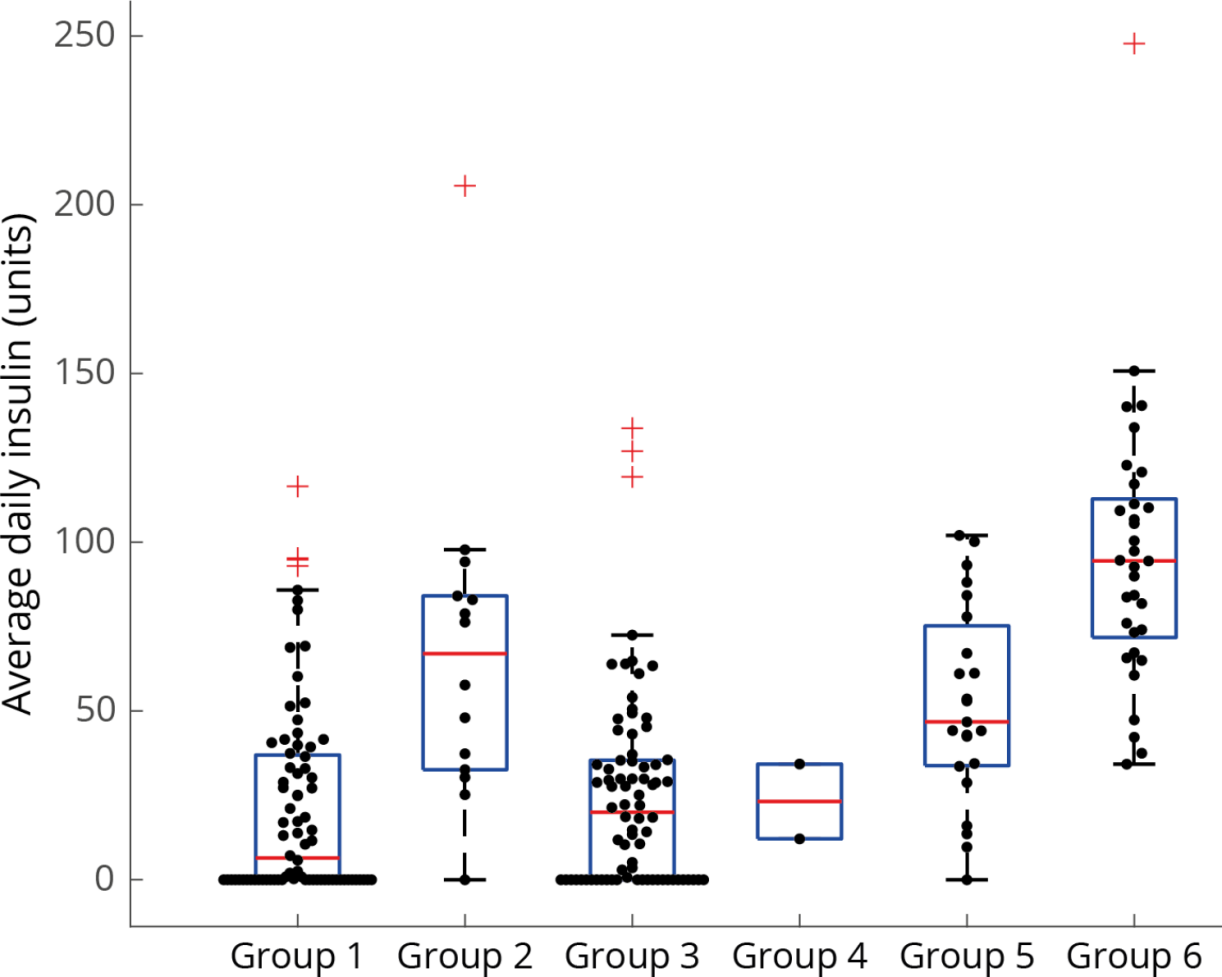
Insulin units per day from intubation onwards, according to predefined strata. Group 1: wave 1, no previous diagnosis of diabetes mellitus. Group 2: wave 1, previously diagnosed diabetes mellitus. Group 3: wave 2, no previous diagnosis of diabetes mellitus, low HbA1c. Group 4: wave 2, previously diagnosed diabetes mellitus, low HbA1c. Group 5: wave 2, no previous diagnosis of diabetes mellitus, high HbA1c. Group 6: wave 2, previously diagnosed diabetes mellitus, high HbA1c. In wave 1, no HbA1c was measured. High HbA1c is defined as above 48mmol/mol (6.5%)

Figure 2 shows descriptive data on the development of serial glucose concentrations and Fig. 3 shows mean insulin units per day for the six predefined strata (i.e., wave 1 without [1] and with [2] diabetes mellitus; the second wave without [3] and with [4] diabetes mellitus with low HbA1c and without [5] and with [6] diabetes mellitus with high HbA1c).

### Associations between serial dysglycemia over time and ICU mortality

ICU non-survivors had higher mean glucose per day of 0.67 (0.25; 1.10) mmol/l (11.2 mg/dl ) 3.6; 19.1) per day as compared to ICU-survivors, which decreased over time with − 0.06 (-0.08; 0.04) mmol/l.

(-1.08 (-1.44; 0.72) mg/dl) per day (Table 2; Model 2). After adjustment for age, sex, BMI, APACHE II score, chronic kidney-, pulmonary- and liver diseases, and cardiovascular risk factors, results showed a similar association over time (Table 2; Model 2). The effect of glucose concentrations on ICU-survival was modified by wave (wave one vs. wave 2) (p-interaction = 0.029), but not by known history of diabetes mellitus (p-interaction = 0.96) or HbA1c (high vs. low) (p-interaction 0.964). Mean glucose results stratified per wave, in adjusted models 2, showed for wave one that ICU non-survivors had a higher mean glucose per day of 0.74 (0.24; 1.23) mmol/l (13.3 (4.3; 22.1) mg/dl) as compared to ICU-survivors, which decreased over time with − 0.04 (-0.06; -0.02) mmol/l per day (-0.72 (-1.08;-0.36) mg/dl); whereas for wave 2 ICU non-survivors had a numerically higher mean glucose per day of 0.37 (-0.25; 0.98) mmol/l (6.7 (4.5; 17.6) mg/dl) as compared to ICU-survivors, which decreased over time with − 0.06 (-0.09; -0.03) mmol/l (-1.08 (-1.62; -0.54 mg/dl)) per day without statistical significance.

From intubation onwards, ICU non-survivors had a greater maximum glucose difference of 3.1979 ± 2.6298 mmol/l (57.6 vs. 57.3 mg/dl) survivors having a maximum glucose difference of 2.6461 ± 2.2318 mmol/l (47.6 vs. 40.1 mg/dl). Non-survivors had a greater maximum glucose difference per day of 0.86 (0.38; 1.34) mmol/l (15.5 (6.8; 24.1 mg/dl) over time as compared to ICU-survivors, which decreased over time with − 0.03 (-0.05; -0.01) mmol/l (-0.54 (-0.09; -0.18) mg/dl) per day (Table 2; Model 1). Adjustment for age, sex, BMI and APACHE II score, chronic kidney, pulmonary and liver diseases, and cardiovascular risk factors resulted in a similar association over time (Table 2; Model 2). The effect of glucose concentration variability on ICU-survival was not shown to be modified by wave (wave 1 vs. 1 wave 2) (p-interaction = 0.199), known history of diabetes mellitus (p-interaction = 0.282) or HbA1c (high vs. low) (p-interaction 0.254).

From intubation onwards, over time, insulin units per day did not differ between ICU non-survivors and ICU survivors in adjusted models (p = 0.755), neither in those with diabetes mellitus, whether known or unknown history nor in those with diabetes mellitus.

**Table 2:** Associations between serial glycemic parameters over time and ICU mortality

	mean glucose					maximum glucose difference				
**Model 1: Crude**	β mmol/l	CI (95%) mmol/l	β mg/dl	CI (95%) mg/dl	p-value	β mmol/l	CI (95%) mmol/l	β mg/dl	CI (95%) mg/dl	p-value
ICU-survivors (reference)	-	-			**-**	-	-			**-**
Mortality	0.67	0.25; 1.10	12.1	4.5; 19.8	**0.002**	0.86	0.38; 1.34	15.4	6.84; 24.12	**< 0.001**
time * ICU non-survivors (slope)	-0.06	-0.08; -0.04	-1.08	-1.44; 0.72	**< 0.001**	-0.03	-0.05; -0.01	0.54	-0.9; -0,18	**0.001**
Model 2*: Adjusted										
ICU-survivors (reference)	-	-	-	-	**-**	-	-	-	-	**-**
Mortality	0.64	0.21; 1.07	11.5	3.8; 19.3	**0.003**	0.87	0.39; 1.35	15.7	7.02; 24.3	**< 0.001**
time * ICU non-survivors (slope)	-0.06	-0.08; -0.04	-1.1	-1.44; -0.72	**< 0.001**	-0.03	-0.05; -0.01	-0.54	0.9; 0.18	**0.007**

β (95% CI) indicates the regression coefficient, or difference and slope over time of mean glucose (mmol/l and mg/dl) and maximum glucose (in mmol/l and mg/dl ) difference per day, respectively, for ICU non-survivors, with ICU survivors as reference. Model 1: Crude analysis. Model 2: adjusted for age, sex, body mass index (BMI) and APACHE II score, chronic kidney, pulmonary and liver diseases, and cardiovascular risk factors

CI: Confidence Interval, ICU: Intensive Care Unit;

*Data on BMI (n = 3) and cardiovascular risk factors (n = 3) were missing; hence model 2 included 226

## Discussion

This prospective study of critically ill COVID-19 patients with comprehensive serial data has four main findings. First, in our study, diabetes mellitus and previously unknown diabetes mellitus were highly prevalent. Second, we showed that non-survivors had higher mean glucose levels and higher maximum differences in glucose concentrations per day during ICU stay compared to survivors. These associations were independent of age, sex, BMI, APACHE II score, chronic kidney, pulmonary, and liver diseases, and cardiovascular risk factors. However, the association between mean glucose and survival weakened and was no longer significant during the second compared to the first COVID-19 wave. Third, we found no evidence to support our hypothesis that the presence of known and previously unknown diabetes mellitus (by high HbA1c) or steroid use worsens glycemic variability associated with prognosis. Finally, total insulin dosage did not differ between survivors and non-survivors, irrespective of diabetes mellitus status, in this cohort of critically ill COVID-19 patients.

Glucose concentrations and glucose variability are independent risk factors for ICU and hospital mortality among various ICU populations [14–19]. The prevalence of known and previously unknown diabetes mellitus in severe COVID-19 is high and associated with a poor prognosis due to glucose dysregulation and other risk factors such as obesity, hypertension [42, 43] and possibly attributable to microvascular abnormalities, rendering them more susceptible after COVID infection to complications or mortality. Furthermore, recent studies on critically ill COVID-19 patients showed that those with glucose concentrations between 3.9 and 10.0 mmol/l had lower mortality than those with higher glucose concentrations [7] and high fasting glucose concentrations [5, 6]. In addition, data on COVID-19 patients admitted to the general ward showed that fasting glucose variability is associated with poor outcome [26, 27, 44]. However, in these studies, only fasting glucose concentrations were used in the first week [44], the first two days [27], or the first three days [26] of general admission. Our study extends these observations by showing adverse effects of 24-h glucose variability on ICU survival that decreased over time. Therefore, we establish glucose variability as a biomarker of dismal prognosis in COVID-19 in ICU.

From this perspective, it is somewhat unexpected that we observed that HbA1c had no interaction with the association between high glucose variability and mortality. However, it should be acknowledged that an HbA1c below 48mmol/mol (6.5%) does not exclude diabetes mellitus [45–47]. This could have had a possible diluting effect on the results of disease outcome. Nevertheless, we observed similar results for a history of known diabetes mellitus not influencing the association between higher maximum glucose difference per day and mortality. Thus, we found no evidence that diabetes mellitus, whether known or previously unknown, based on high HbA1c, and a diagnosis of diabetes mellitus, leads to unfavorable outcomes independent of glycemic parameters/dysglycemia. Alternatively, the observation that glucose variability, as reflected by daily maximum glucose difference, is associated with mortality may also be explained by the suggestion that glucose concentrations are suggested as a biomarker of systemic inflammation, whereas HbA1c is a proxy of glucose control in the past three months [8].

We found no evidence to support our hypothesis that steroid use worsens glycemic variability-induced prognosis. Mean glucose concentrations were higher in non-survivors compared to survivors, which is in line with earlier findings that hyperglycemia worsens prognosis in ICU-populations [10, 11, 19] and COVID-19 [5–7]. Furthermore, this association was only present in the pre-steroid era wave (1) Thus, even though steroids exposed more patients to hyperglycemia, any association between mean glucose concentration and mortality was weaker rather than stronger (hence losing statistical significance) in wave (2) These observations suggest that the beneficial effects of steroids on mortality in COVID-19 seem to outweigh the harmful effects of steroids on glucose control in this cohort and aligns with a previous observational study which reported steroids to be associated with dysglycemia in COVID-19 but did not have a significant association with 30-day mortality [23]. Despite the aforementioned, maximum glucose difference is shown to be a strong determinant of worse outcome in previous studies in general critical care, regardless of pre-existing diabetes mellitus [40]. Increased glycemic variability has been studied before and found to be associated with worse outcome in terms of mortality and length of stay in various ICU and non-ICU populations [48–50] However, since we observed an association between higher maximum glucose difference per day and higher mortality, independent of known risk factors, comorbidities, without effect-modification by wave and known or unknown diabetes mellitus, we provide evidence to focus supportive care on in order to ameliorate survival of critically ill COVID-19 patients.

Daily total insulin dosage was administered following a standard ICU regimen. This variable not being statistically significantly different between survivors and non-survivors could be due to our relative insensitive approach to lump total insulin dose within one day, in contrast to an hour-to-hour insulin variability. However, we had no hour-to-hour data on insulin, which is a limitation of our study. It could also be that insulin dosage has considerable confounding by illness severity precluding the study of direct beneficial/harmful effects of insulin itself. Previous work by Uyttendaele and colleagues however found higher insulin sensitivity in non-survivors, whereas hour-to-hour insulin variability was equivalent in both non-survivors and survivors among a mixed-medical ICU population, suggesting equal controllability [51]. Perhaps not directly generalizable to the present severe COVID-19 population, our results in the perspective of the previous findings by Uyttendaele strengthen the importance of improving glucose control in critical care.

We need to address some limitations. First, the study is a single-center study. It is observational, so no conclusions with regard to causality can be drawn. Next, we used pandemic waves as a proxy of steroid use as steroids became standard of care and were protocoled in the Netherlands from wave 2 onwards. This per-protocol approach allowed for investigating effect modification in an interpretable way as adding daily dexamethasone data would be more complicated, and due to the protocolized administration of dexamethasone to all wave 2 ICU-admitted COVID-19 patients, an intention-to-treat approach would likely not change our results. Another limitation that requires addressing is the absence of information regarding specific diabetes treatment (such as SGLT2 inhibitors that aside from diabetes mellitus, can have other indications including heart failure) and diabetic complications at the time of inclusion when a diagnosis of diabetes was present. Furthermore, glucose measurements were conducted in accordance with established standards of care, albeit without adherence to a predefined and standardized study protocol, which could have introduced its own sources of variability. Furthermore, we used HbA1c for diagnostic purposes since it is a reliable measure of chronic dysglycemia. However, a value less than 48 mmol/mol (or 6.5%) does not completely exclude diabetes mellitus diagnosed using glucose tests [46, 47]. For future studies, it would be interesting to see which glycemic patterns our population had in terms of fasting and non-fasting glucose concentrations before their admission to the intensive care unit. At last although we did not have information on whether patients had type 1 or type 2 diabetes mellitus, in light of the biometric and comorbid attributes it is highly likely that the predominant diabetes type is 2. The strengths of our study are the prospective and extensively phenotyped cohort having systematic data collection performed using a predefined protocol [28]. Furthermore, we provide serial glucose measurements daily, which is very informative in providing measures of glucose variability.

## Conclusions

In conclusion, known and unknown history of diabetes mellitus were often present in patients with COVID-19 admitted to the ICU. Non-survivors had significantly higher daily maximum glucose differences throughout their ICU stay compared to patients with COVID-19 that survived their ICU stay. This effect was independent of age, sex, BMI and APACHE II score, chronic kidney, pulmonary, and liver diseases, and cardiovascular risk factors but was not modified by a history of diabetes mellitus or dexamethasone use during ICU stay. Our results point toward preventing hyperglycemia and large glucose variability in critically ill COVID-19 patients in the ICU.

### List of abbrevations

SARS-CoV-2: Severe Acute Respiratory Syndrome Coronavirus 2; HbA1c: glycated haemoglobin; ICU: intensive care unit; MaastrICCht: Maastricht Intensive Care COVID cohort study; CORADS: COVID-19 Reporting and Data System; eCRF: electronic case report form; STROBE: Strengthening the Reporting of OBservational studies in Epidemiology; Apache II: Acute Physiology and Chronic Health Evaluation; BMI: body mass index;

## Data Availability

The datasets generated during and/or analyzed during the current study are available from the corresponding author on reasonable request.
